# Olfaction Modulates Inter-Subject Correlation of Neural Responses

**DOI:** 10.3389/fnins.2020.00702

**Published:** 2020-07-10

**Authors:** Paul DeGuzman, Anshul Jain, Matthias H. Tabert, Lucas C. Parra

**Affiliations:** ^1^Neuromatters, LLC, New York, NY, United States; ^2^International Flavors & Fragrances, Inc., R&D, Union Beach, NJ, United States; ^3^Department of Biomedical Engineering, City College of New York, New York, NY, United States

**Keywords:** inter-subject correlation (ISC), electroencephaloagraphy (EEG), attention, reliability, fragrance, olfaction

## Abstract

Odors can be powerful stimulants. It is well-established that odors provide strong cues for recall of locations, people and events. The effects of specific scents on other cognitive functions are less well-established. We hypothesized that scents with different odor qualities will have a different effect on attention. To assess attention, we used Inter-Subject Correlation of the EEG because this metric is strongly modulated by attentional engagement with natural audiovisual stimuli. We predicted that scents known to be “energizing” would increase Inter-Subject Correlation during watching of videos as compared to “calming” scents. In a first experiment, we confirmed this for eucalyptol and linalool while participants watched animated autobiographical narratives. The result was replicated in a second experiment, but did not generalize to limonene, also considered an “energizing” odorant. In a third, double-blind experiment, we tested a battery of scents including single molecules, as well as mixtures, as participants watched various short video clips. We found a varying effect of odor on Inter-Subject Correlation across the various scents. This study provides a basis for reliably and reproducibly assessing effects of odors on brain activity. Future research is needed to further explore the effect of scent-based up-modulation in engagement on learning and memory performance. Educators, product developers and fragrance brands might also benefit from such objective neurophysiological measures.

## Introduction

The flavors and fragrance industry has traditionally focused on the discovery and blending of raw ingredients to create taste and scent profiles for a wide range of product categories. While the process to extract, synthesize, formulate and deliver fragrance materials has advanced over the decades, the focus and methods for consumer testing remain largely unchanged – relying on creative briefs from brands, professional scent design, expert and technical evaluations, and the self-report ratings of sensory and naïve consumer panels to substantiate desired sensorial attributes and performance (Mattei et al., 2015).

In response to a growing demand from consumers the flavors and fragrance industry is increasingly focused on delivering proven functional benefits related to health and wellness that go beyond traditional approaches such as Aromatherapy (Hongratanaworakit, 2004) or anecdotal evidence (e.g., “lavender oil is good for sleep” or “peppermint oil is energizing”).

The general effects of odors on cognitive function have been studied intensively (see Table 1). However, the evidence for effects of specific fragrances is sparse. Anecdotal evidence from aromatherapy and academic studies have shown that select essential oils can have a positive impact on cognitive functions, e.g., attention, alertness, learning and memory (e.g., Rasch et al., 2007; Moss and Oliver, 2012; Moss et al., 2014). However, few studies, if any, have systematically characterized the functional benefits of a broader range of scent materials that include both synthetic molecules and mixtures. Our goal is to identify objective neurophysiological measures of the effects of individual scents on cognitive function.

**TABLE 1:** Summary of studies showing function benefits of scent exposure.

**Topic**	**References**
Smoking cessation	Arzi et al., 2014
Olfaction perception in sleep	Carskadon and Herz, 2004
Memory	Diekelmann and Born, 2010
Learning	Stickgold, 2012
Learning	Arzi et al., 2012
Clerical tasks associated with administration	Barker et al., 2003
Alertness and math computations	Diego et al., 1998
Anxiety reduction	Redd et al., 1994
Severity of labor pain	Yazdkhasti and Pirak, 2016
Pain tolerance	Prescott and Wilkie, 2007

The effects of olfactory stimulation on brain activity have been well-characterized (Herz et al., 2004; Osterbauer et al., 2005; Gottfried et al., 2006; Li et al., 2008; Plailly et al., 2008; Howard et al., 2009), but the specific effects of different fragrances is not well-established (Brauchli et al., 1995; Martin, 1998; Lorig, 2000; Kroupi et al., 2014). In the current study, we employed a validated, cost-effective and practical approach based on electroencephalography (EEG). Traditional EEG research has largely employed event-related designs, which analyzes the immediate (<1 s) response after stimulus presentation. Due to the slower speed of delivery and sensory processing it has been difficult to do the same with odors (Lorig et al., 1993, 1999). Other approaches use oscillatory EEG activity which fluctuate on a slower time scale. For example, alpha activity (10 Hz oscillation) fluctuates in power on a scale of 10 s, and is known to be modulated by attention (Klimesch, 2012) but there are no consistent reports of olfaction on alpha activity (Lorig, 2000).

Instead, here we rely on a recent finding that EEG evoked activity can be significantly correlated between subjects while they watch naturalistic video stimuli, such as TV shows (Dmochowski et al., 2012), video advertising (Dmochowski et al., 2014), YouTube clips (Cohen and Parra, 2016), or movie trailers (Barnett and Cerf, 2017). Correlation of brain activity across subjects measured with functional MRI has been linked to memory and efficacy of communication (Hasson et al., 2008; Honey et al., 2012; Zadbood et al., 2017). Importantly, the level of this Inter-Subject Correlation in the EEG (ISC) is strongly modulated by attention (Ki et al., 2016) and is predictive of memory (Cohen and Parra, 2016), learning (Cohen et al., 2018; Bevilacqua et al., 2019) and audience retention (Cohen et al., 2017). The presumed mechanism for this in EEG is that attention increases evoked response magnitude and this in turn increases ISC (Ki et al., 2016; Poulsen et al., 2017). The main conclusion of this work is that ISC can be used as a marker of attentional engagement with naturalistic stimuli. In other words, ISC measures how alert subjects are as they perceive their natural environment. Here we propose to use ISC as an objective measure of the effects of scents on attentional engagement. Our specific hypothesis is that ISC of the EEG is modulated by exposure to fragrances during the viewing of a video narrative. In this work, we test this hypothesis directly with the goal to establish the ISC of the EEG as an objective measure for the impact of fragrances on alertness.

## Results

### Overview of Experiments and Objectives

Experiment 1 was a pilot experiment to test the effects of two odorants relative to a no-odor control. We used eucalyptol, considered an “energizing” odor (Moss and Oliver, 2012), and linalool, considered “calming” (Kuroda et al., 2005; Höferl et al., 2006). The primary outcome measure was ISC of the stimulus-evoked response while participants watched short animated autobiographical narratives (StoryCorps/Modern Love) video clips (see Cohen and Parra, 2016). The goal was to determine whether attentional engagement with the naturalistic audiovisual stimulus can be modulated by concurrent presentation of contrasting scents. The secondary outcome measures were traditional event related potentials and power of oscillatory activity.

Experiment 2 was a single-blind replication and optimization study, which sought to determine whether the results depended on the specific video clips used and what signal duration was necessary to obtain robust results. Here we used eucalyptol and limonene to test if the effect on ISC generalizes to other “energizing” odors such as limonene (Heuberger et al., 2001; Herz, 2009). Analysis focused on the primary outcome measures of ISC.

Experiment 3 was a double-blind screening study with 10 odors covering mixtures and single molecules with varying degrees of trigeminal stimulation (Doty et al., 1978). The goal was to test whether we obtain a differential effect of odors on ISC. Both participants as well as experimenters were blind to the nature or identity of the test odorants.

In all experiments, the videos were counterbalanced so as to appear equally often in all odor conditions. In all cases, ISC was compared with values within subjects to remove random variability across subjects.

### Experiment 1: Eucalyptol Enhances ISC but Has No Effect on Odor-ERP or Alpha Power

**FIGURE 1:**
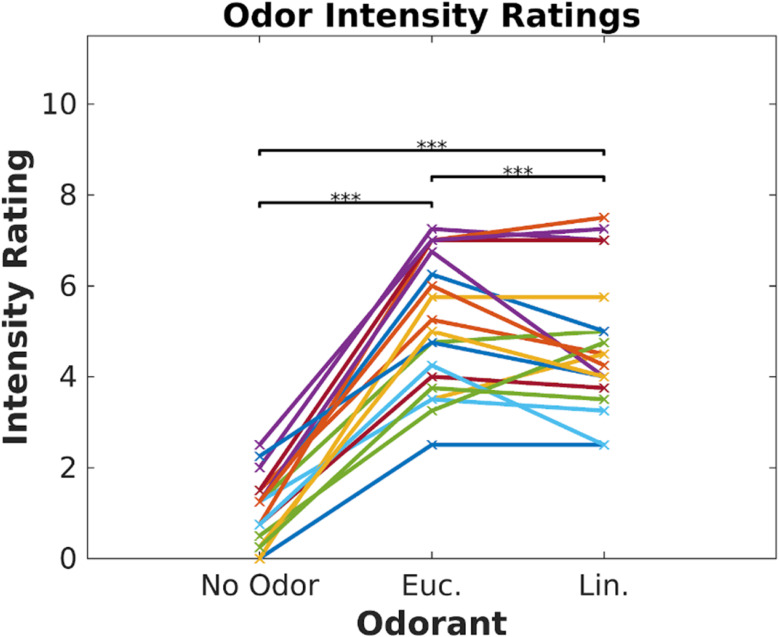
Odor intensity was equalized for Eucalyptol and Linalool. Ratings of perceived intensity for the 2 odorants and the no-odor control in Experiment 1. Each participant is shown as a line and values are the mean of 4 repeated ratings per participant. The result of pairwise testing for significance is indicated as *p < 0.05, **p < 0.01, ***p < 0.001.

Participants (*N* = 19) watched 6 video clips (2–3 min each, Cohen and Parra, 2016) in two blocks of three videos. Each block was paired with one of three odor conditions (eucalyptol, linalool and no-odor vehicle), repeating each block three times such that all videos were paired with all odor conditions. Order of video blocks and odors were couterbalanced across subjects to control for temporal order effects. To prevent habituation, odors were presented in brief 6 s bursts repeated every 30 s (Tabert et al., 2007). The concentrations of the odorants were adjusted prior to the experiment to provide similar subjective intensity. To verify this, all subjects were asked to rate on a scale of 0 to 10 the “intensity” of each odor at the beginning of the experiment (Figure 1). Both odorants were perceived to be stronger than the no-odor control [eucalyptol: *t*(18) = 13.66, *p* < 10^–9^; linalool: *t*(18) = 11.5, *p* < 10^–9^]; all p values in this paper are two sided and computed with a paired *t*-test except where otherwise stated), but no different from one-another in intensity [*t*(18) = 1.71, *p* = 0.10]. Therefore, any difference we may find in the neural responses cannot be attributed to differences in perceived odorant intensity.

**FIGURE 2:**
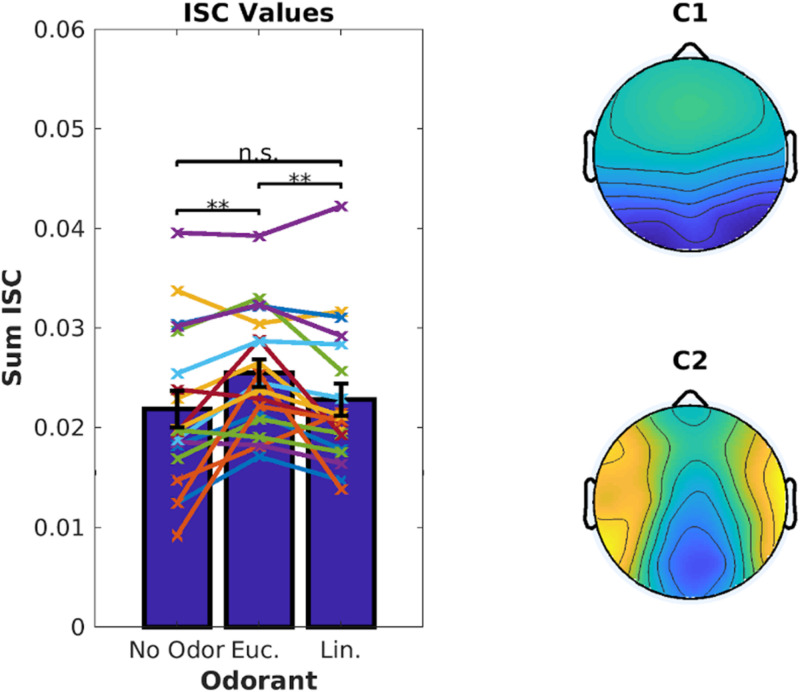
Inter-Subject Correlation of the EEG during video presentation is enhanced by Eucalyptol, but not Linalool. Inter-Subject Correlation (ISC) is measured by combining signals from multiple EEG electrodes and measuring the correlation coefficient of the time courses between one subject and all others exposed to the same video clips. **Left:** ISC values for individual participants averaged across all videos for the 3 odor conditions shown as line graphs. The corresponding average values are shown as bars. **Right:** The combination of electrodes is reflected in components with a spatial distribution shown here across the scalp. These top two EEG components are maximally correlated between the participants.

Inter-Subject Correlation of the stimulus-evoked EEG was measured using established techniques (Parra et al., 2018). We computed ISC values averaged over the 6 video clips corresponding to 18.7 min of EEG data. There was a significant increase in ISC when the videos were presented with eucalyptol as compared to both linalool and the no-odor control [Figure 2, left; linalool: *t*(18) = 3.05, *p* = 0.007, no-odor: *t*(18) = 3.64, *p* = 0.0019]. The EEG components that capture the largest correlation between subjects (Figure 2, right) were similar to previous results obtained for these video stimuli in the absence of olfactory stimulation (Cohen and Parra, 2016). This suggests that the increase in ISC is the result of a modulation of visual and auditory activity elicited by the audiovisual stimulus, rather than odor-evoked responses. It is interesting to note that both eucalyptol and linalool are trigeminal in nature (Doty et al., 1978), but only eucalyptol up-modulated ISC. Therefore, it is unlikely that the modulation is caused by an overall increase in alertness caused by trigeminal stimulation alone.

#### No Effect of Odors on ERP or Oscillatory Power

**FIGURE 3:**
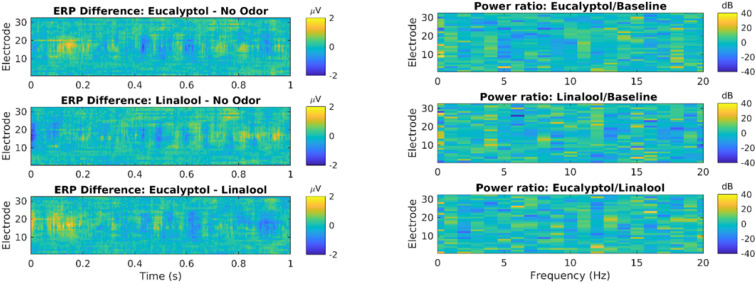
Odor event-related potentials and oscillatory power are not significantly modulated by Eucalyptol of Linalool relative to no-odor control. **Left:** Event related potential (ERP) elicited by odor presentation, shown here for all electrodes as a time resolution of 2 ms. ERP is the average over all odor exposure events (722 odor presentations events from 19 subjects). Here the difference in ERP between different odor conditions is shown. Time is measured relative to odor onset. Statistical testing showed no significant difference (shuffle statistics, *N* = 1000 randomizations, only ∼1% of time-electrode bins cross a *p* < 0.01 threshold). **Right:** Power of oscillations of the EEG was analyzed in each electrode and different frequency bands. Shown are the log-power difference in the 1 s following odor exposure for different electrodes and frequency bins (0.5 Hz). No statistical testing was performed as there are no evident differences in power.

We also measured traditional event related potentials (ERPs) following the onset of odor exposure (Figure 3, left). Statistical testing of all electrodes and time delays up to 1 s after odor onset show no significant difference between odors and controls (*N* = 722 events in each condition; no time/electrode combination passes *p* = 0.05 after FDR correction for 32^∗^256 comparisons). We also measured oscillatory power in that same time period, but now resolved by frequency between 0 and 20 Hz (Figure 3, right). There was no evident effect at any frequency or electrode. Thus, in this pilot study, we did not see an effect of odors on ERPs or oscillatory power, the two most conventional outcome metrics for EEG.

### Experiment 2: Reliability and Scalability

**FIGURE 4:**
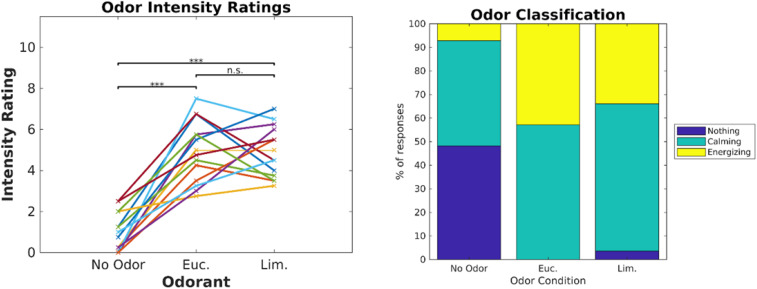
Eucalyptol and Limonene were equalized in intensity and were similarly rated as “calming” or “energizing.” **Left:** Individual ratings of perceived intensity for the 2 odorants and the no-odor control in Experiment 2. **Right:** Percent of participants that perceived the odorants as calming, energizing or neutral. The result of pairwise testing for significance is indicated as *p < 0.05, **p < 0.01, ***p < 0.001.

Our ultimate goal was to perform routine biometric testing on a large number of fragrances and scents. Thus, the objective of Experiment 2 was to determine if the modulation of ISC depended on the specific videos used and whether we could use shorter video stimuli. We used the same six videos as in Experiment 1, but fixed the presentation order (Figure 9). Participants (*N* = 14) were exposed to eucalyptol and limonene as a second “energizing” odor (Heuberger et al., 2001; Herz, 2009) with low levels of trigeminal nerve activation at moderate intensity levels (Doty et al., 1978). Participants rated the two odors similarly in terms of odor intensity [Figure 4, left; (*t*(13) = 0.039, *p* = 0.97]. We also asked participants to categorize odors as “calming” or “energizing” (Figure 4, right). Both odors were judged in similar proportions as calming and energizing.

**FIGURE 5:**
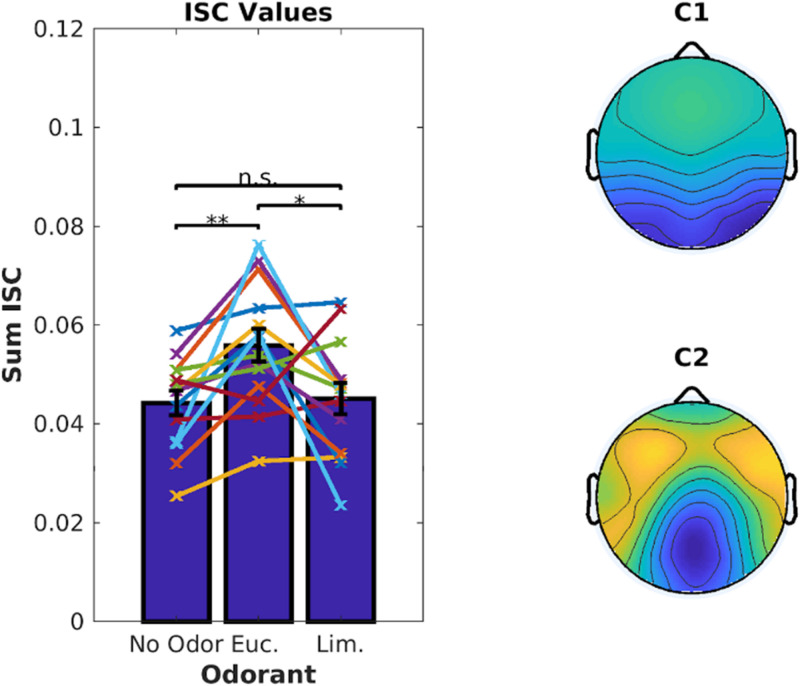
Eucalyptol enhanced ISC but not Limonene. **Left:** ISC values for individual participants averaged across all 6 videos for the 3 odor conditions. Each line represents a participant. The corresponding average values are shown as bars. **Right:** The top 2 EEG components that are maximally correlated between the participants. They are similar to those in Figure 2. Result of pairwise testing for significance indicated as *p < 0.05, **p < 0.01, ***p < 0.001.

We first computed ISC combining all 6 video clips with ∼18 min of data per odor condition. As before, eucalyptol up-modulated participants’ ISC compared to the no-odor control [Figure 5, left, *t*(13) = 3.85, *p* = 0.002]. Limonene did not increase ISC over the no-odor control [*t*(13) = 0.41, *p* = 0.69], and was lower than eucalyptol [*t*(13) = 2.62, *p* = 0.021]. This replicates the results of Experiment 1 for eucalyptol but does not apply to limonene which is traditionally considered to be energizing. Therefore, a boost in ISC should not be considered a measure of “energizing” effects of odors in the traditional sense of the aromatherapy literature.

**FIGURE 6:**
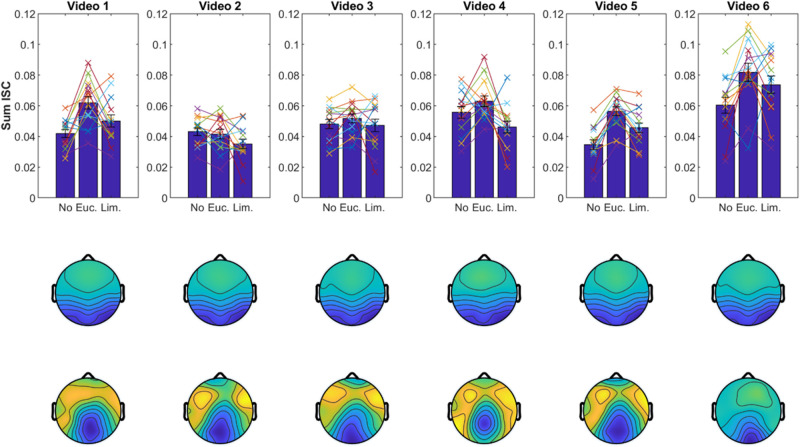
Results replicate with shorter data segments. Same display as in Figure 4 but ISC values and correlated components are computed separately for each video clip (∼3-min duration each).

We obtained similar results when we computed ISC with shorter data segments of ∼3 min from individual videos (Figure 6). The spatial distribution of the corresponding correlated components was also well-preserved, which further attests to the robustness of results with shorter data segments. To test if the choice of video clip has an effect on the modulation of ISC, we performed a three-way ANOVA (with subject as a random effect). As expected, we found again an effect for odor [*F*(2) = 6.2, *p* = 0.0024]. We also found an effect for video [*F*(5) = 23.8, *p* < 10^–9^] indicating that some videos elicited higher ISC than others, but we found no interaction between video and odor [*F*(10) = 1.15, *p* = 0.334]. This suggests that odors modulate ISC similarly for different video clips. Although, given the shorter segments, one should note that on individual videos the expected trend is not preserved (e.g., video 2; Figure 6).

Finally, we repeated the oscillatory power analysis (as in Figure 3, right) and found no differences in power between Eucalyptol and Limonene relative to one another or relative to no-odor anywhere between 0 to 20 Hz (not shown).

### Experiment 3: Differential Effect of a Battery of Odors on ISC

The results from Experiment 1 and 2 showed that odor based modulation of ISC can be reliably measured over a period of 3 min. Experiment 3 was designed to extend these findings to odors with a wide range of odor character, trigeminal quality and complexity (mixtures vs single molecules). Each participant (*N* = 20) watched a video clip paired with one odor, and again in a separate session on a separate day without the odor (see methods). The pairing of the 10 video clips with the 10 odorants was randomized, and the order of the odor/no-odor condition counterbalanced across the two sessions. Neither participants, experimenters or data analysts were aware of the name or nature of these odorants.

**FIGURE 7:**
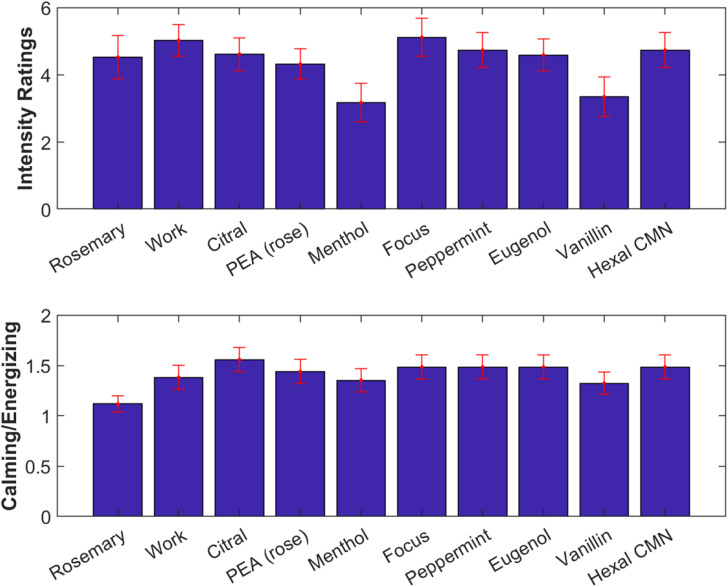
All odorants tested were subjectively similar in intensity and quality. **Top:** Subjective intensity ratings averaged over all participants. Error bars (red) indicate standard error of the mean. **Bottom:** Ratio of number of participants rating odorants as calming vs energizing.

Participants were again asked to rate odor intensity and rate them as “energizing” or “calming” (Figure 7). A one-way repeated measures ANOVA found no difference in the subjective intensity ratings across odors [*F*(9) = 0.93, *p* = 0.50, Figure 7, top]. Generally, odorants were judged more often as calming, and this did not significantly differ between odorants [*F*(9) = 0.46, *p* = 0.90, Figure 7, bottom].

**FIGURE 8:**
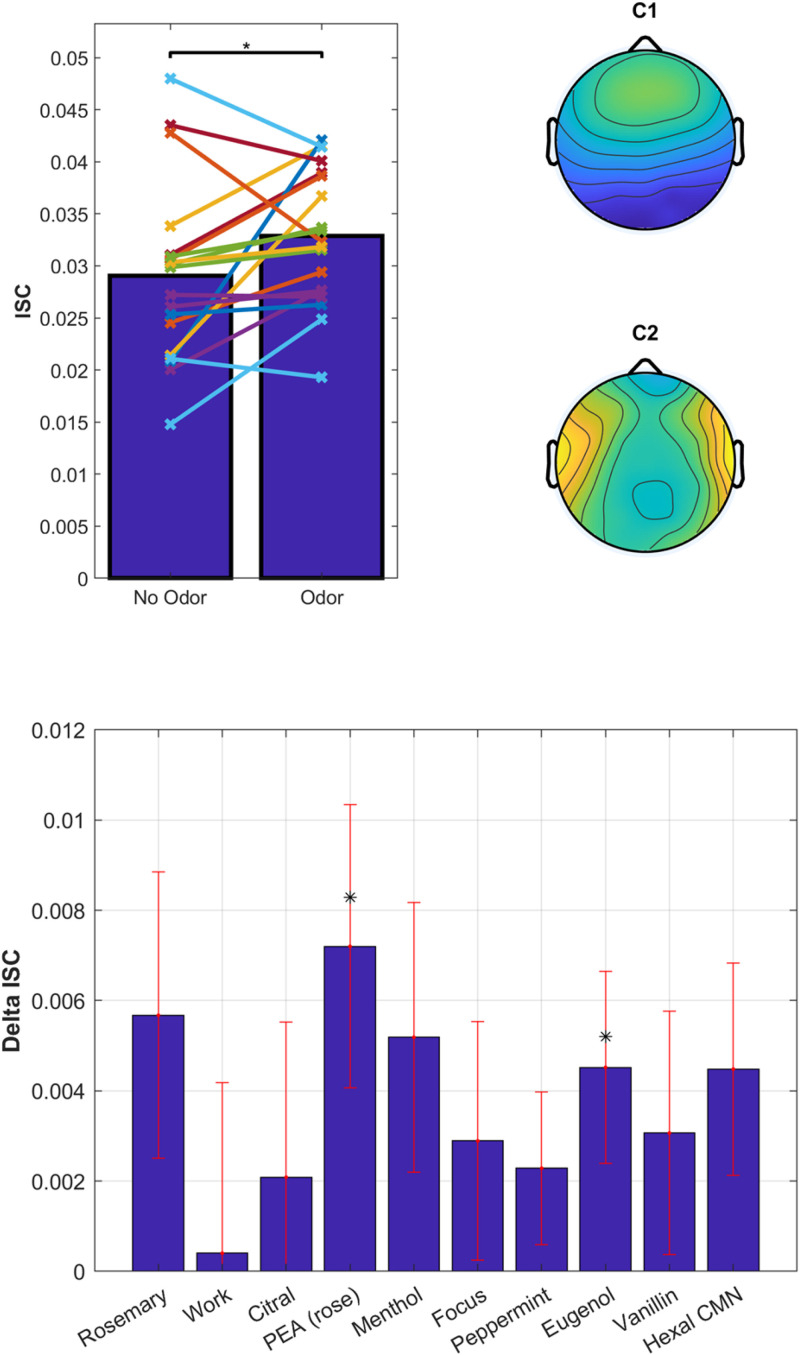
Odorants have a differential effect on ISC of the EEG elicited by audiovisual stimuli. **Top:** ISC values averaged over all odorants and non-odorant conditions. Each line represents a participant (*N* = 20). Spatial distribution of the EEG components with the largest correlation suggestive of visual and auditory processing. **Bottom:** Delta ISC (odor minus non-odor) averaged across subjects. Error bars (red) indicate standard error of the mean.

Overall, the odors enhanced ISC compared to no-odor control [Figure 8, top; *t*(18) = 2.2, *p* = 0.038]. There appears to be a difference between odorants on the degree to which they modulated ISC (Figure 8, bottom). However, a one-way repeated-measures ANOVA on the change in ISC does not resolve this effect [odor – non-odor: *F*(9) = 0.67, *p* = 0.73]. We found strongest modulation for Phenyl ethyl alcohol (PEA, rose) followed by Rosemary Oil and Eugenol, with a significant modulation for PEA [*t*(18) = 2.3, *p* = 0.034] and Eugenol [*t*(18) = 2.12, *p* = 0.048]. Interestingly, both PEA and Eugenol are considered to have low trigeminal effects at moderate perceived intensity levels, while Citral and Peppermint are known to have pronounced trigeminal effects (Doty et al., 1978). This further suggests that trigeminal stimulation is not driving the observed effects. Furthermore, the primary component of Rosemary Oil is Eucalyptol, which means that the up modulation with Eucalyptol has been replicated across 3 different experiments with 3 different sets of participants; lending further support to the robustness of this effect and our ability to reliably measure it.

## Discussion

In summary, we have shown that Inter-Subject Correlation of the EEG evoked by narrative auditory-visual stimuli is reliably and reproducibly modulated by olfactory stimuli and that this modulation is dependent on the particular odorant being presented. We have further shown that in odors across a range of olfactive qualities, this modulation is not dependent on the nature of the video, trigeminal nature of the odorant (Table 2), or whether the odorant is categorized as energizing or calming.

**TABLE 2:** List of odorants tested in Experiment 3.

**Odorant**	**Category**	**Dosage (%)**	**Trigeminal**
Peppermint oil	Essential oil	0.3	Yes
Rosemary oil	Essential oil	0.3	Yes
Citral refined	Molecule	1	Yes
Fragrance focus	Mixture	0.3	Yes
Fragrance work	Mixture	0.3	Yes
Hexyl Cinn Ald	Molecule	15	No
Vanillin	Molecule	0.3	No
Phen Ethyl Alc	Molecule	0.1	No
Eugenol USP	Molecule	0.3	No
Menthol	Molecule	3	Yes

While we replicated results for eucalyptol in each of the 3 studies, the differences between 10 different odors could not be reliably resolved with a sample of 20 subjects. We ascribe this to the variability observed in ISC across subjects. For a systematic ranking of a larger number of odors, we thus recommend larger sample sizes. Subsequent studies may explore the reliability of odor ranking as a function of sample size. On the flip side, we do not believe that one requires as many electrodes as have been used here (*N* = 32). In practice, it may suffice to use 16 or even 8 electrodes, provided they are strategically placed. An additional caveat is the selection of videos. Here we used a set of 10 different short video clips to keep participants interested in the material. We do know that different stimuli will elicit different levels of ISC. By controlling with a no-odor condition with the same video clip we may have reduced some of this variability. However, it is possible that using different video material does add variability to the study. Future studies that seek to use ISC on a routine basis to evaluate odors may need to parametrize the dependence of the video stimuli used and possibly standardize the results.

The components with maximal ISC observed in this series of experiments are similar to those we observed previously with purely audiovisual stimulation (e.g., Cohen et al., 2017), suggesting that odorants modulate auditory-visual processing rather directly driving the neural response. This is confirmed by the lack of a direct observation of odor event-related potentials. The components of the EEG extracted with the present technique during video watching are known to be multisensory (Cohen and Parra, 2016). The spatial distributions of these components on the scalp are very similar to those earlier studies. Multisensory components that are broadly distributed are difficult to localize with EEG (if nothing else, because inverse modeling in EEG is an ill-posed problem). Nonetheless, earlier fMRI studies have attempted at localizing these components (Dmochowski et al., 2014) and identify correlated fMRI activity in the superior temporal sulcus likely due to auditory processing, as well as activity in the precuneus and anterior cingulate, which have been interpreted in that earlier study as self-referential processing. Further studies will need to be designed to explore the underlying neural mechanism for the effects observed here, specifically in the context of odors.

We have found here that odors modulate the EEG activity evoked by natural dynamic audiovisual stimuli. This is consistent with existing reports that odors can modulate visual and auditory event-related potentials. For example, odor modulate ERPs evoked by images of faces (Bensafi et al., 2002; Cook et al., 2015; Leleu et al., 2015; Syrjänen et al., 2018). Visual ERPs are also modulated when paired with congruent or incongruent olfactory stimuli (Seo et al., 2010; Robinson et al., 2015). Animal experiments also show a modulation of auditory evoked responses in the presence of odors (Halene et al., 2009). A recent study shows that ERPs in response to fearful images is modulated in infants by the scent of the mother (Jessen, 2019). This phenomenon was explained as an attentional effect (the presence of the mother allows the infant to attend less to threatening stimulus). Attention is well known to modulate the magnitude of auditory and visual evoked responses, this in turn results in increased ISC (Poulsen et al., 2017). Given the known modulation of ISC with attention (Ki et al., 2016), it is likely that varying attention accounts for the modulation observed here with different odors.

Despite this interpretation, one important caveat of the study is that we have used relatively small sample sizes here. Additionally, we have no behavioral readout of attention and therefore cannot draw strong conclusions in this regard. An alternative explanation is that the stimuli were physiologically arousing (Cuthbert et al., 2000) and this affected evoked response magnitudes without affecting attention. Ultimately, the blinded randomized sham-controlled trials we have performed here can only conclude that odors had an effect on ISC.

We analyzed alpha power given its established link to attention during sensory processing. Typically, alpha power is attenuated over sensory cortices during active and attentive processing of a visual or auditory stimulus (Foxe and Snyder, 2011). Yet, we did not find an effect of odor on alpha power. Given our interpretation of the effect of odor on ISC as a modulation of attention, this null result is particularly surprising. We had previously established that attention modulates both ISC of evoked responses as well as alpha power (Ki et al., 2016). But, the effect size of attention on alpha is quite a bit weaker than on ISC. It is possible that here, the attentional modulation was not sufficient to affect a measurable modulation of alpha power. In fact, ISC modulation is relatively weak here. An alternative explanation is that the effect of odor on audiovisual evoked response magnitude is direct (as discussed above). In that scenario, odors might not modulate attention toward the stimulus, but directly modulate sensory evoked responses.

We also did not find a modulation of potentials evoked by the odor itself, i.e., odor-evoked ERPs. In our reading of the literature, the odor-evoked ERP is relatively weak (Lorig et al., 1993, 1999). It may be that we did not collect long enough records or a sufficient number of subjects to resolve an attentional effect on what is already a weak ERP signal.

Given the extensive literature linking ISC modulation to attention and engagement with a natural audiovisual stimulus, we conclude that olfaction can have a differential modulatory effect on how alert we are when perceiving the natural world in a holistic and multisensory context. Importantly, previous studies have shown that ISC values while watching educational videos predict learning performance as measured by performance on a follow-up test like questionnaire (Cohen et al., 2018), or that subjects with higher ISC have better recall of episodic memories (Cohen et al., 2017). In future studies, we plan to explore if up-modulation in engagement driven by odors results in a similar increase in learning and memory performance. In doing so, Inter-Subject Correlation may help educators to improve attention and cognitive performance of students, as well as brands, to substantiate marketing claims made on the functional benefits of fragrances, which currently have to rely on more subjective measures of mood and emotion, or more broadly defined cognitive constructs and dimensions such as “energizing” and “calming.”

## Materials and Methods

### Stimulus Presentation, Data Collection and Analysis

For all experiments, the odors were delivered via a modified OLFACT^TM^ (Osmic Enterprises, Inc., Cincinnati, OH, United States) olfactometer. The delivery of the odor was synchronized with the respiration cycle as measured by a nasal flow pressure transducer (SleepSense Product Code: SS-14833/E). The odor was delivered for 6 s at the onset of an inhalation phase with a 24 s inter-stimulus-interval (ISI) (Tabert et al., 2007).

32-channel EEG, fitted in accordance with the 10/20 standard layout, and nasal respiration were recorded with a BioSemi ActiveTwo System (BioSemi, Amsterdam, Netherlands) at a sampling frequency of 2,048 Hz. The audio-visual stimuli were presented via Psychtoolbox (Brainard, 1997; Pelli, 1997; Kleiner et al., 2007) on a flat screen computer monitor.

EEG data pre-processing steps followed the procedure described in previous studies (Cohen and Parra, 2016) in order to remove eye-movement artifacts. Outlier samples, defined as values exceeding three times the distance between the 25th and 75th quartile of the median centered signal, were identified within each channel and replaced with zero-valued samples including 40 ms of signal before and after the outlier samples.

#### Inter-Subject Correlation

ISC calculations for each condition followed an identical procedure to that described in previous studies (Cohen and Parra, 2016; Parra et al., 2018). Briefly, signals are linearly combined across electrodes to form “components.” These component signals are correlated across time between subjects. Each subject pair provides a correlation coefficient, and these are then averaged over all subject pairs involving one participant. Therefore, for each participant and component, there is one ISC value. These are then summed over the strongest correlated components to arrive at Sum ISC values. The linear weights for each component are derived from the data by maximizing Sum ISC computed for all available data. When reporting Sum ISC per odor, we use only data during that odor presentation. ISC was calculated using the sum of top two components of EEG signal that maximally capture the correlated responses across participants. In earlier studies, we used 3 or more components (Dmochowski et al., 2012, 2014; Cohen and Parra, 2016; Ki et al., 2016; Cohen et al., 2017, 2018). Here, however, in experiment 1 we found that the higher components (3 and higher) were not consistent across odors and therefore decided to limit to the first two for the first and subsequent experiments.

As in previous studies there is significant variation in ISC across subjects. We control for this by measuring only the change in ISC, e.g., difference to no-odor, or difference between odors. This means that we are doing within-subject control by using a paired t-test or, equivalently, repeated-measures ANOVA with subject as random effect.

### Study Stimuli

#### Odor Samples

For Experiment 1, Eucalyptol was diluted to 10% in Triethyl Citrate (CAS# 77-93-0). Other oils used in Experiment 1 and 2 (Linalool and Limonene) were neat oils. All presented stimuli were presented at iso-intense levels (Figures 1, 4, 7). Triethyl Citrate was presented by itself as a no-odor control.

For Experiment 3, the ten materials shown in Table 2 were presented at concentrations to ensure iso-intensity with those presented in Experiment 1.

#### Video Clips

For Experiments 1 and 2, the same set of six video clips were presented (Table 3). All videos were professionally produced and of engaging narrative content and previously used in ISC studies (Cohen and Parra, 2016). For Experiment 3, there were 10 groups of videos used, where each of the 10 pairs of videos had a total duration of at least 6 min (Table 4).

**TABLE 3:** Videos used in Experiment 1 and Experiment 2.

**Vid ID**	**Duration**	**URL**
1	3:17	https://www.youtube.com/watch?v=okF5UGpivR8
2	3:56	https://www.youtube.com/watch?v=yfWa9gI-Bks
3	3:03	https://www.youtube.com/watch?v=yAC-z8F0Mdw
4	3:17	https://www.youtube.com/watch?v=iGnCvLPZm84
5	3:02	https://www.youtube.com/watch?v=Nf3MM7jzkZw
6	2:07	https://www.youtube.com/watch?v=6m85l_UqM5I

**TABLE 4:** Videos used in Experiment 3.

**Vid Group**	**Vid ID**	**Duration**	**URL**	**Vid ID**	**Duration**	**URL**	**Total duration**
A	1	3:17	https://www.youtube.com/watch?v=okF5UGpivR8	11	2:48	https://www.youtube.com/watch?v=gvdIaLHYUws	6:05
B	2	3:56	https://www.youtube.com/watch?v=yfWa9gI-Bks	12	2:33	https://www.youtube.com/watch?v=bW82E98FBCA	6:29
C	3	3:03	https://www.youtube.com/watch?v=yAC-z8F0Mdw	13	3:15	https://www.youtube.com/watch?v=ZPIaVilHr5M	6:18
D	4	3:17	https://www.youtube.com/watch?v=iGnCvLPZm84	14	2:44	https://www.youtube.com/watch?v=jZK7rayEptw	6:01
E	5	3:02	https://www.youtube.com/watch?v=Nf3MM7jzkZw	15	3:33	https://www.youtube.com/watch?v=YvDtOigTH-g	6:35
F	6	2:07	https://www.youtube.com/watch?v=6m85l_UqM5I	16	3:59	https://www.youtube.com/watch?v=eO7sKVKMO2s	6:06
G	7	2:22	https://www.youtube.com/watch?v=KtGbpTOk844	17	3:39	https://www.youtube.com/watch?v=KQF79ch6mA8	6:01
H	8	2:07	https://www.youtube.com/watch?v=Tlk4ocdavm0	18	3:56	https://www.youtube.com/watch?v=iRpKjghGQec	6:03
I	9	2:04	https://vimeo.com/76641635	19	4:18	https://www.youtube.com/watch?v=xSKuOccVVKg	6:22
J	10	1:34	https://www.youtube.com/watch?v=XypF2zV_LVs	20	5:51	https://www.youtube.com/watch?v=WNfvuJr9164	7:25

*There were 10 video groups which consisted of a pair of videos totaling to a duration of at least 6 min.*

### Study Participants

For each study, a separate group of young male and female participants between 18 and 24 years old were recruited and provided written informed consent (Table 5). All participants self-reported normal ability to smell and had no known allergies to scent materials. All data collection and procedures were approved by Western Institutional Review Board (WIRB).

**TABLE 5:** Subject demographics per experiment.

**Experiment**	**Total**	**Number of female**	**Age (mean)**	**Age (SD)**
1	19	11	20.2	3.6
2	14	8	20.6	2.8
3	20	13	20.9	2.1

### Experimental Design

All experiments followed a within-subject design. For Experiment 1, participants watched all 6 videos under all odor conditions – linalool, eucalyptol & no-odor condition. The videos were presented in blocks of three videos, with only one odor presented in any given block. The order of videos within each block, as well as the order of blocks for a given odorant, was randomized between participants. The presentation order of odorants was counterbalanced between participants. All participants evaluated the intensity of the odors on a 0–10 scale at the beginning of the session.

**FIGURE 9:**
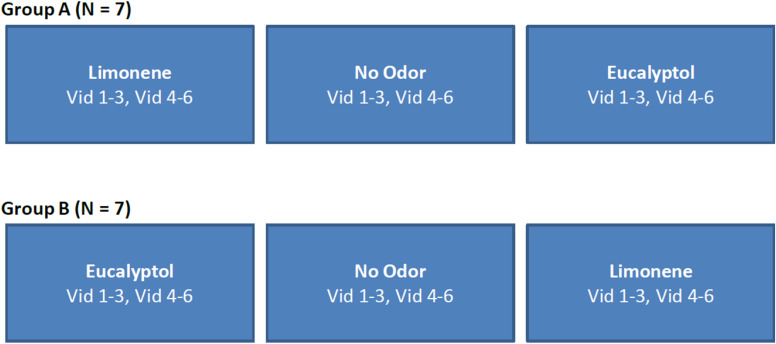
Study design used in Experiment 2. The order of videos was fixed and counterbalanced across participants. The no-odor control condition was always presented in-between the two odor conditions in order to minimize any carry over effects.

For Experiment 2 the same six videos as in Experiment 1 were used, but the order in which the videos were presented was fixed (Figure 9). This allowed us to perform ISC analysis on a subset of the videos to determine minimum length needed for a reliable response (results not presented), as well as determine if the delta (odor vs no-odor) depends on the specific films selected. All other aspects of the experiment design were identical to Experiment 1.

For Experiment 3, a set of 10 video groups were selected (each group consisted of a pair of videos totaling to a duration of at least 6 min) and tested in the presence of 10 odors (see Table 2). Here, a Latin Square Williams design was used so that each video group and olfactory stimulus was paired with each other and all video groups were presented with a no-odor control. The order of odors and video groups were pseudo-randomized and counterbalanced across participants. The complete design is shown in Table 6. The data was collected over two 60-min sessions for each participant.

**TABLE 6:** Latin Square Williams design in Experiment 3.

	**Vid Group 1**	**Vid Group 2**	**Vid Group 3**	**Vid Group 4**	**Vid Group 5**	**Vid Group 6**	**Vid Group 7**	**Vid Group 8**	**Vid Group 9**	**Vid Group 10**
Subject 1	1	2	3	4	5	6	7	8	9	10
Subject 2	2	3	4	5	6	7	8	9	10	1
Subject 3	10	1	2	3	4	5	6	7	8	9
Subject 4	3	4	5	6	7	8	9	10	1	2
Subject 5	9	10	1	2	3	4	5	6	7	8
Subject 6	4	5	6	7	8	9	10	1	2	3
Subject 7	8	9	10	1	2	3	4	5	6	7
Subject 8	5	6	7	8	9	10	1	2	3	4
Subject 9	7	8	9	10	1	2	3	4	5	6
Subject 10	6	7	8	9	10	1	2	3	4	5
Subject 11	1	2	3	4	5	6	7	8	9	10
Subject 12	2	3	4	5	6	7	8	9	10	1
Subject 13	10	1	2	3	4	5	6	7	8	9
Subject 14	3	4	5	6	7	8	9	10	1	2
Subject 15	9	10	1	2	3	4	5	6	7	8
Subject 16	4	5	6	7	8	9	10	1	2	3
Subject 17	8	9	10	1	2	3	4	5	6	7
Subject 18	5	6	7	8	9	10	1	2	3	4
Subject 19	7	8	9	10	1	2	3	4	5	6
Subject 20	6	7	8	9	10	1	2	3	4	5

*Additionally, each video was presented to each participant under a no-odor control condition. Therefore, a participant watched the same video twice – once with odor and once without odor.*

## Data Availability Statement

The datasets generated for this study are available on request to the corresponding author.

## Ethics Statement

The studies involving human participants were reviewed and approved by Western Institutional Review Board (WIRB). The patients/participants provided their written informed consent to participate in this study.

## Author Contributions

LP, PD, AJ, and MT designed the experiment. PD performed the research. PD and LP analyzed the data. LP, PD, AJ, and MT wrote the mansucript. All authors contributed to the article and approved the submitted version.

## Conflict of Interest

The authors declare that this study received funding from International Flavors & Fragrances, Inc. The funder had the following involvement with the study through the contributions of their employees (AJ and MT): conducted iso-intensity testing, provided scent stimuli, provided guidance with scent delivery and contributed to article writing. PD was employed by Neuromatters, LLC. The remaining author declares that the research was conducted in the absence of any commercial or financial relationships that could be construed as a potential conflict of interest.
